# The chimeric antibody chLpMab-7 targeting human podoplanin suppresses pulmonary metastasis via ADCC and CDC rather than via its neutralizing activity

**DOI:** 10.18632/oncotarget.5339

**Published:** 2015-09-25

**Authors:** Yukinari Kato, Akiko Kunita, Shinji Abe, Satoshi Ogasawara, Yuki Fujii, Hiroharu Oki, Masashi Fukayama, Yasuhiko Nishioka, Mika K. Kaneko

**Affiliations:** ^1^ Department of Regional Innovation, Tohoku University Graduate School of Medicine, Aoba-ku, Sendai, Miyagi 980-8575, Japan; ^2^ Department of Pathology, Graduate School of Medicine, the University of Tokyo, Bunkyo-ku, Tokyo 113-0033, Japan; ^3^ Department of Clinical Pharmacy Practice Pedagogy, Institute of Biomedical Sciences, Tokushima University Graduate School, Tokushima 770-8505, Japan; ^4^ Department of Respiratory Medicine and Rheumatology, Institute of Biomedical Sciences, Tokushima University Graduate School, Tokushima 770-8503, Japan

**Keywords:** podoplanin, PDPN, metastasis, monoclonal antibody, ADCC/CDC

## Abstract

Podoplanin (PDPN/Aggrus/T1α) binds to C-type lectin-like receptor-2 (CLEC-2) and induces platelet aggregation. PDPN is associated with malignant progression, tumor metastasis, and poor prognosis in several types of cancer. Although many anti-human PDPN (hPDPN) monoclonal antibodies (mAbs), such as D2-40 and NZ-1, have been established, these epitopes are limited to the platelet aggregation-stimulating (PLAG) domain (amino acids 29-54) of hPDPN. Recently, we developed a novel mouse anti-hPDPN mAb, LpMab-7, which is more sensitive than D2-40 and NZ-1, using the Cancer-specific mAb (CasMab) method. The epitope of LpMab-7 was shown to be entirely different from that of NZ-1, a neutralizing mAb against the PLAG domain according to an inhibition assay and lectin microarray analysis. In the present study, we produced a mouse-human chimeric anti-hPDPN mAb, chLpMab-7. ChLpMab-7 showed high antibody-dependent cellular cytotoxicity (ADCC) and complement-dependent cytotoxicity (CDC). Furthermore, chLpMab-7 inhibited the growth of hPDPN-expressing tumors *in vivo*. Although chLpMab-7 recognizes a non-PLAG domain of hPDPN, it suppressed the hematogenous metastasis of hPDPN-expressing tumors. These results indicated that chLpMab-7 suppressed tumor development and hematogenous metastasis in a neutralization-independent manner. In conclusion, hPDPN shows promise as a target in the development of a novel antibody-based therapy.

## INTRODUCTION

Podoplanin (PDPN/Aggrus/T1α) is a platelet aggregation-inducing type I transmembrane *O*-glycoprotein [1–3]. The expression of human PDPN (hPDPN; 162 amino acids) has been reported in many cancers, including oral cancers, malignant brain tumors, esophageal cancers, lung cancers, malignant mesotheliomas, bladder cancers, testicular tumors, and osteosarcomas [2, 4–16]. The expression of hPDPN in cancer-associated fibroblasts (CAFs) has been associated with poor prognosis in patients with cancer [17–21]. We previously compared the migration activities of PDPN-transfected osteosarcoma cells and parental cells and found that PDPN-transfected osteosarcoma cells exhibited a higher migration activity [14]. In solid tumors such as brain tumors, only a small and phenotypically distinct subset of cells can be responsible for generating and sustaining tumors, and these cells are considered cancer stem cells [22]. Because cancer stem cells are thought to be resistant to conventional therapies and are responsible for relapse, targeting cancer stem cells may provide a promising approach to cancer therapy [23]. PDPN has been reported to be a cancer stem cell marker [24]; therefore, immunotherapy using specific antibodies against hPDPN may eradicate cancer stem cells in cancers.

PDPN has been reported to possess important physiological functions. For the embryonic separation of blood-lymphatic blood vessels, interaction with PDPN-C-type lectin-like receptor-2 (CLEC-2) and platelet aggregation are critical [25]. We recently performed crystallographic studies of hPDPN bound to CLEC-2 [26]. The interaction with CLEC-2 was primarily observed at Glu47 and Asp48 in the platelet aggregation-stimulating (PLAG) domain (amino acids 29–54) and the α2–6 linked sialic acid at Thr52 of hPDPN. The development of ectopic lymphoid follicles is also dependent on PDPN, which is expressed in Th17 cells [27]. PDPN binds to CLEC-2 and rearranges the actin cytoskeleton in dendritic cells to promote efficient mobility along stromal surfaces [28]. The signaling pathway triggered by the interaction of PDPN with CLEC-2 controls the contractility of fibroblastic reticular cells and lymph node microarchitecture [29]. CLEC-2-expressing dendritic cells control the tension of the PDPN-expressing fibroblastic reticular network and lymph node expansion [30].

In this study, we investigated whether a novel mouse-human chimeric anti-hPDPN monoclonal antibody (mAb), chLpMab-7, shows ADCC and CDC activities against hPDPN-possessing tumors, inhibits the growth of hPDPN-expressing tumors, and suppresses hematogenous metastases in a neutralization-independent manner.

## RESULTS

### Characterization of the LpMab-7 epitope

**Figure 1:**
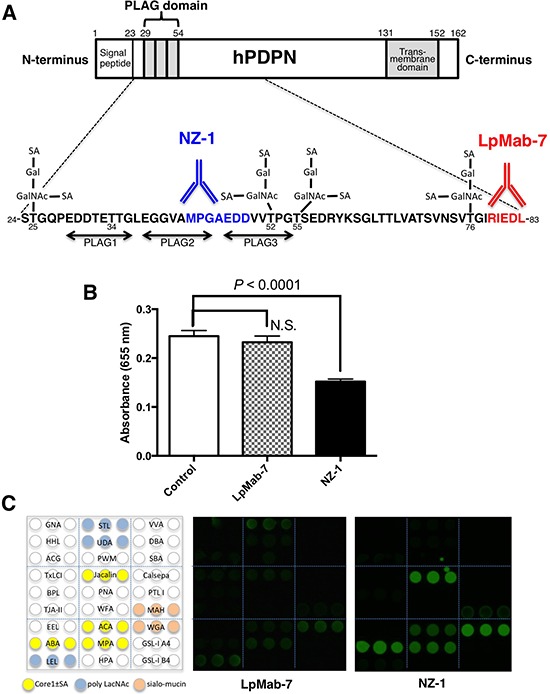
Characterization of the LpMab-7 epitope **A.** Comparison of epitopes of anti-hPDPN mAbs. PLAG, platelet aggregation-stimulating domain; SA, sialic acid; Gal, galactose; GalNAc, *N*-acetylgalactosamine. The minimum epitopes of NZ-1 and LpMab-7 are MPGAEDD and RIEDL, respectively. **B.** The hPDPN-CLEC-2 interaction was blocked by NZ-1 but not by LpMab-7. Inhibition assay was performed using ELISA. Purified hPDPN-Fc was immobilized at 1 μg/ml. After blocking, LpMab-7 or NZ-1 was added at 1 μg/ml. The plates were incubated with biotinylated CLEC-2-Fc (1 μg/ml) followed by 1:1000-diluted peroxidase-conjugated streptavidin. The enzymatic reaction was conducted with a 1-Step Ultra TMB-ELISA. The optical density was measured at 655 nm. **C.** Antibody-overlay lectin microarray. Purified hPDPN was applied to a lectin array. After incubation, human IgG was applied to each well for blocking. Then, biotinylated LpMab-7 and NZ-1 were applied and Cy3-labeled streptavidin was added. The glass slide was scanned using a GlycoStation Reader 1200.

We previously produced a novel anti-hPDPN mAb, LpMab-7 (IgG_1_, kappa) [31], and identified the minimum epitope of LpMab-7 as Arg79-Leu83 of hPDPN (162 amino acids), which is distinct from the PLAG domain [32]. As shown in Figure 1A, the epitope of LpMab-7 is not the PLAG domain, whereas that of NZ-1, a neutralizing mAb, is the PLAG domain. In the present study, we further characterized the epitope of LpMab-7 using inhibition assay and lectin microarray. We first determined whether LpMab-7 inhibits hPDPN-CLEC-2 interaction using ELISA. As a positive control, a rat anti-hPDPN mAb, NZ-1, was used. As shown in Figure 1B, LpMab-7 did not block the binding of CLEC-2-Fc to hPDPN-Fc, whereas NZ-1 significantly inhibited the interaction. Therefore, the epitope of LpMab-7 is entirely different from that of NZ-1. We further performed an antibody-overlay lectin microarray analysis of hPDPN using LpMab-7 and NZ-1 (Figure 1C). LpMab-7 faintly detected sialic acid ± core1 binders (Jacalin, ACA, MPA), weakly detected a sialo-mucin binder (WGA) and moderately detected poly LacNAc binders (LEL, STL). In contrast, NZ-1 strongly detected sialic acid ± core1 binders (ABA, Jacalin, ACA, MPA) and a sialo-mucin binder (WGA) but weakly detected another sialo-mucin binder (MAH). These results indicate that LpMab-7 can be used for detecting different glycan profiles on hPDPN, which has not been observed in anti-PLAG domain mAbs such as NZ-1.

### Production and characterization of a chimeric anti-hPDPN antibody, chLpMab-7

**Figure 2:**
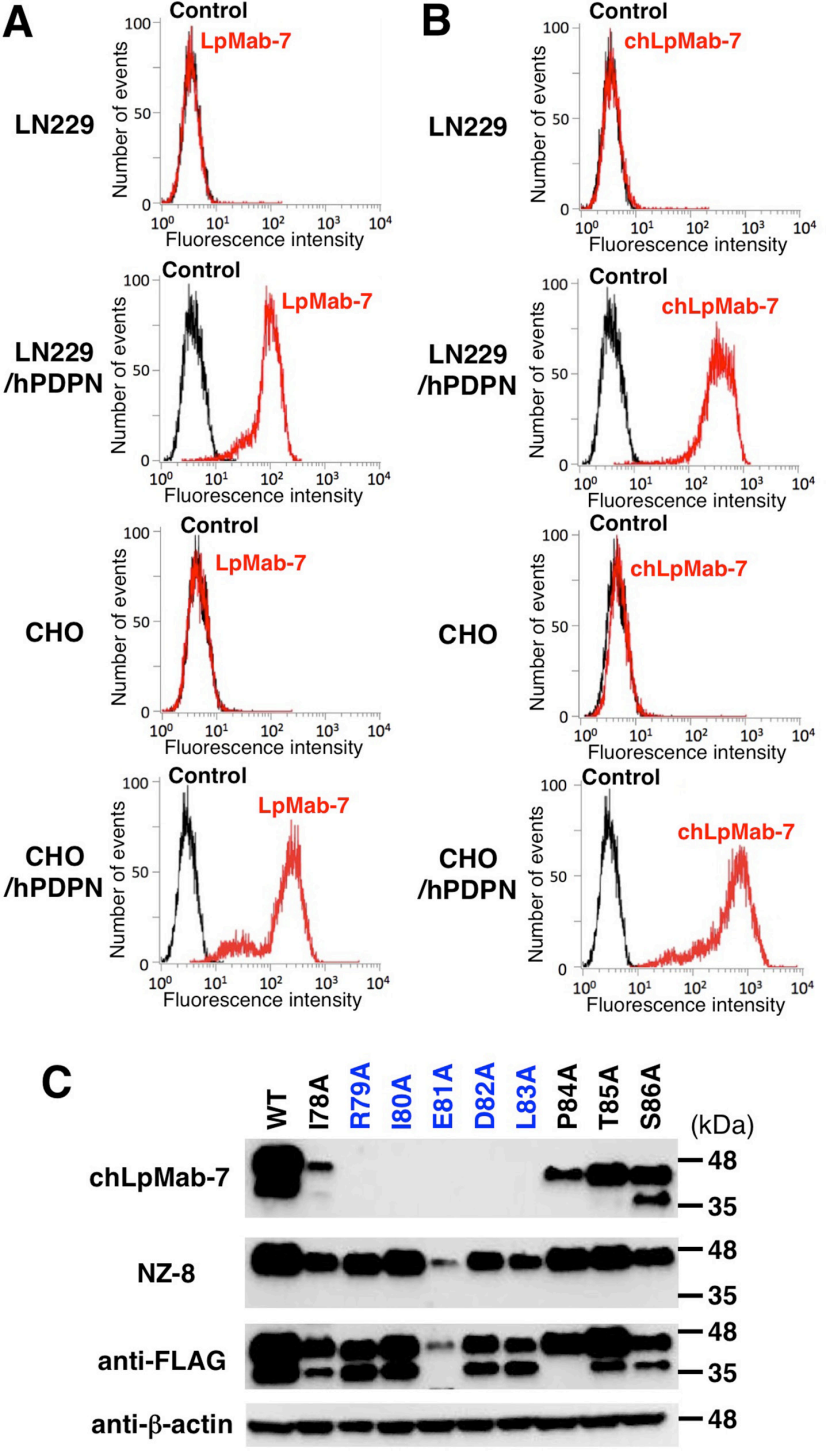
Flow cytometry of hPDPN-transfected cells using anti-hPDPN antibodies LpMab-7 and chLpMab-7 Cells were harvested by brief exposure to 0.25% Trypsin/1 mM EDTA. After washing with PBS, the cells were treated with LpMab-7 **A.** and chLpMab-7 **B.** followed by treatment with secondary antibodies. Fluorescence data were collected using a Cell Analyzer EC800. **C.** The epitope mapping of chLpMab-7 by Western blot analysis. Total cell lysates of point mutants of hPDPN were electrophoresed on 5–20% polyacrylamide gels and transferred onto a PVDF membrane. After blocking, the membrane was incubated with 1 μg/ml mAbs (chLpMab-7 or NZ-8 against hPDPN, 1E5 against FLAG tag, AC-15 against β-actin) and then with peroxidase-conjugated secondary antibodies; the membrane was analyzed using a Sayaca-Imager.

**Figure 3:**
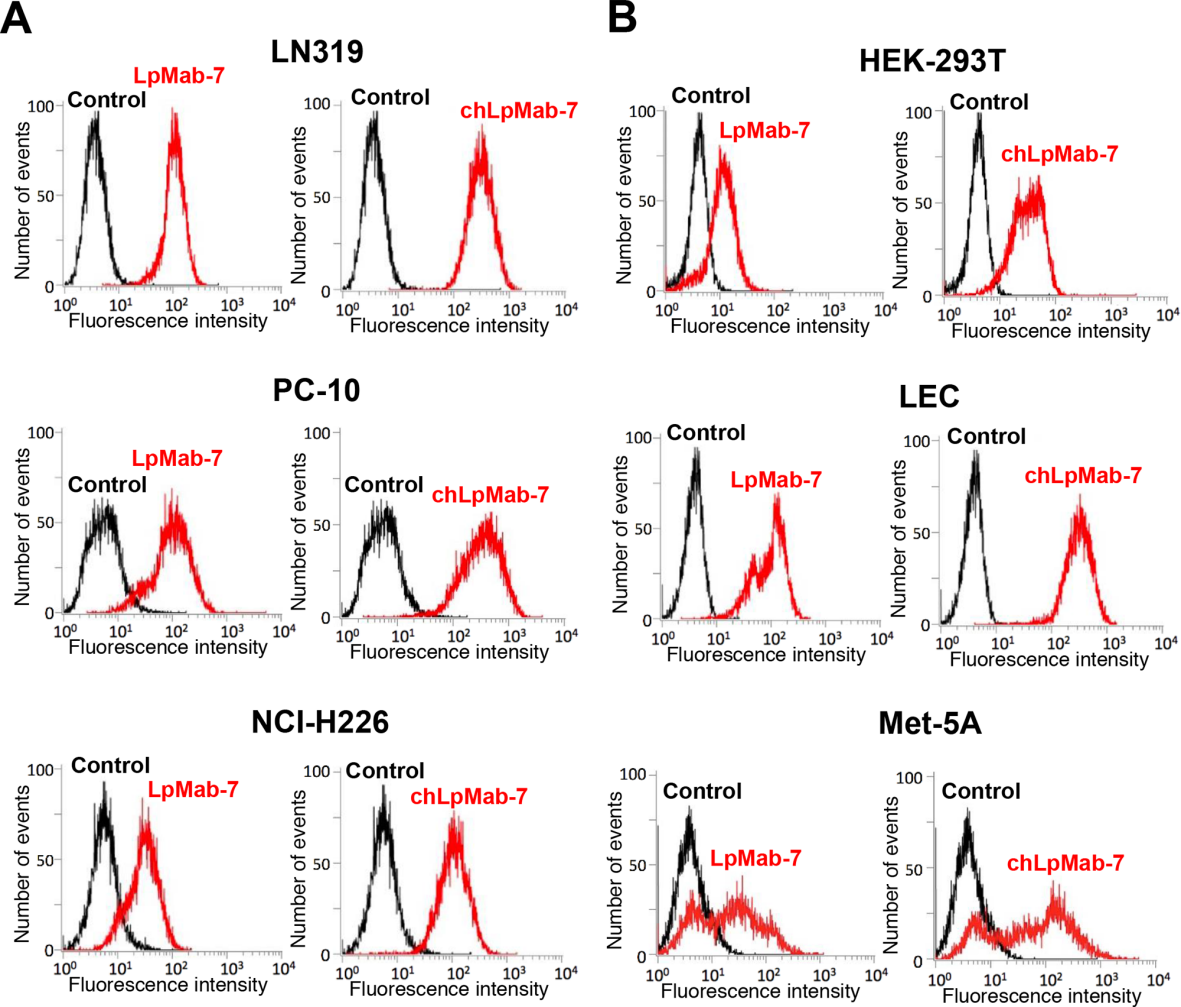
Flow cytometry of endogenous hPDPN-expressing cell lines using anti-hPDPN antibodies LpMab-7 and chLpMab-7 Cancer cells **A.** or normal cells **B.** were harvested by brief exposure to 0.25% Trypsin/1 mM EDTA. After washing with PBS, the cells were treated with LpMab-7 and chLpMab-7 followed by treatment with secondary antibodies. Fluorescence data were collected using a Cell Analyzer EC800.

**Figure 4:**
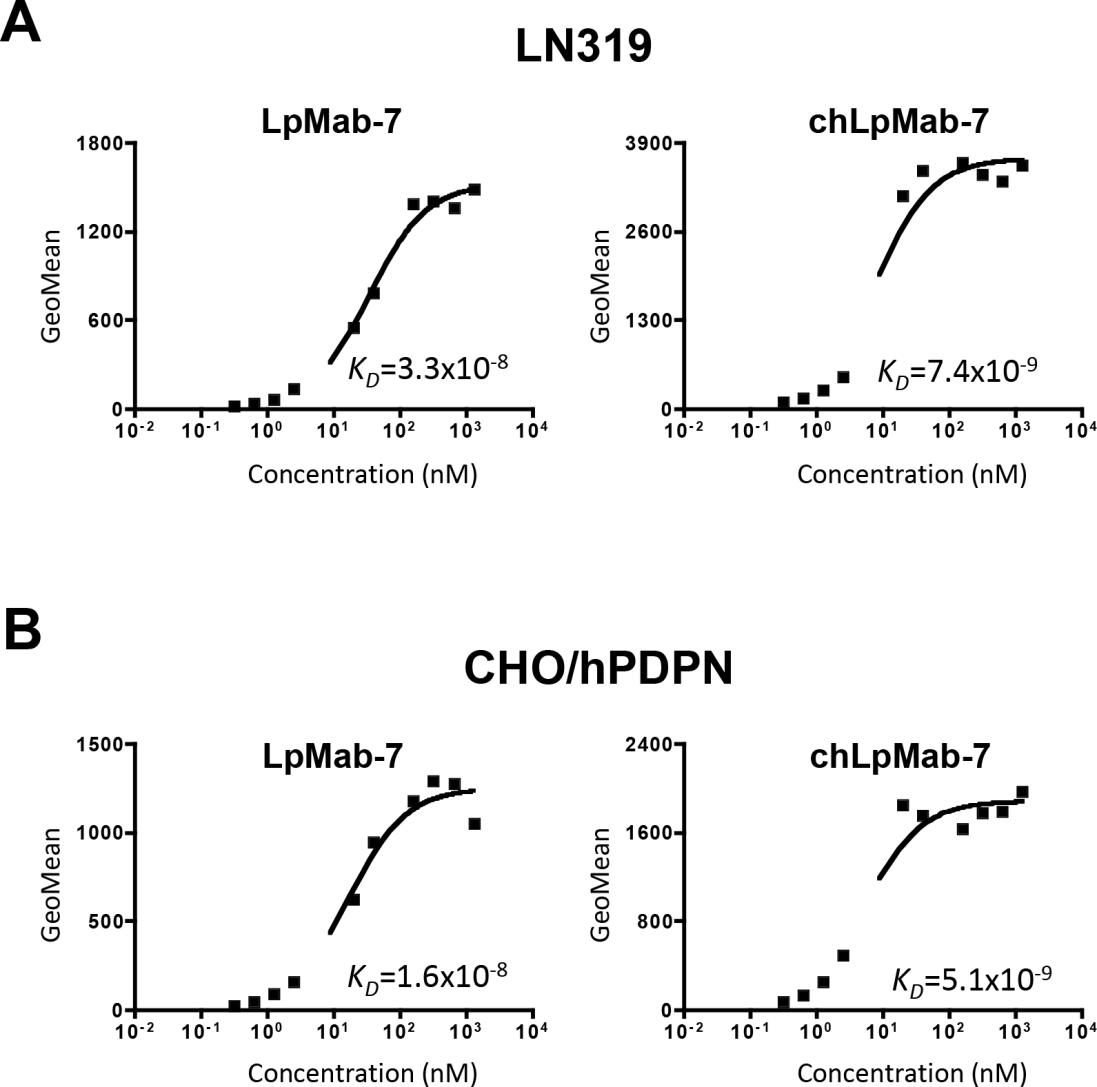
Determination of the binding affinity using flow cytometry LN319 and CHO/hPDPN cells (2 × 10^5^) were resuspended at 100 μl of serially diluted LpMab-7 **A.** or chLpMab-7 **B.** (0.02–100 μg/ml). Fluorescence data were collected using a Cell Analyzer EC800. *K_D_* was obtained by fitting the binding isotherms using the built-in one-site binding models in Prism software.

We next generated a mouse-human chimeric antibody (chLpMab-7) by fusing the V_H_ and V_L_ regions of a mouse mAb (LpMab-7) with the C_H_ and C_L_ regions of human IgG_1_, respectively. Both LpMab-7 and chLpMab-7 recognized hPDPN in LN229/hPDPN and CHO/hPDPN cells, whereas neither mAb reacted with the parental cells (Figure 2A and 2B), indicating that both mAbs specifically target hPDPN. The epitope of chLpMab-7 is Arg79-Leu83 of hPDPN, which is consistent with the epitope of LpMab-7 (Figure 2C). The reaction of LpMab-7 and chLpMab-7 against hPDPN was compared using hPDPN-expressing cancer cell lines and normal cell lines. Previous reports demonstrated that hPDPN is expressed in the human glioblastoma cell line LN319, the human lung squamous cell carcinoma cell line PC-10, the human mesothelioma cell line NCI-H226, the kidney cell line HEK-293T, lymphatic endothelial cells, and the mesothelial cell line Met-5A [7, 9, 31, 33–36]. Both LpMab-7 and chLpMab-7 similarly reacted with cancer cell lines, such as LN319, PC-10, and NCI-H226 and normal cell lines, including HEK-293T, lymphatic endothelial cells, and Met-5A, as revealed by flow cytometry (Figure 3). Because the binding affinity of antibodies is critical for antibody-based cancer therapy, the apparent dissociation constant (*K_D_*) was next determined using flow cytometric analysis. As shown in Figure 4, the *K_D_* of chLpMab-7 was determined to be 7.4 × 10^−9^ M against LN319 cells (Figure 4A) and 5.1 × 10^−9^ M against CHO/hPDPN cells (Figure 4B) by flow cytometry. In contrast, the *K_D_* of LpMab-7 was determined to be 3.3 × 10^−8^ M against LN319 cells (Figure 4A) and 1.6 × 10^−8^ M against CHO/hPDPN cells (Figure 4B), indicating that the binding affinity of chLpMab-7 is greater than that of LpMab-7.

### ADCC and CDC are mediated by chLpMab-7

**Figure 5:**
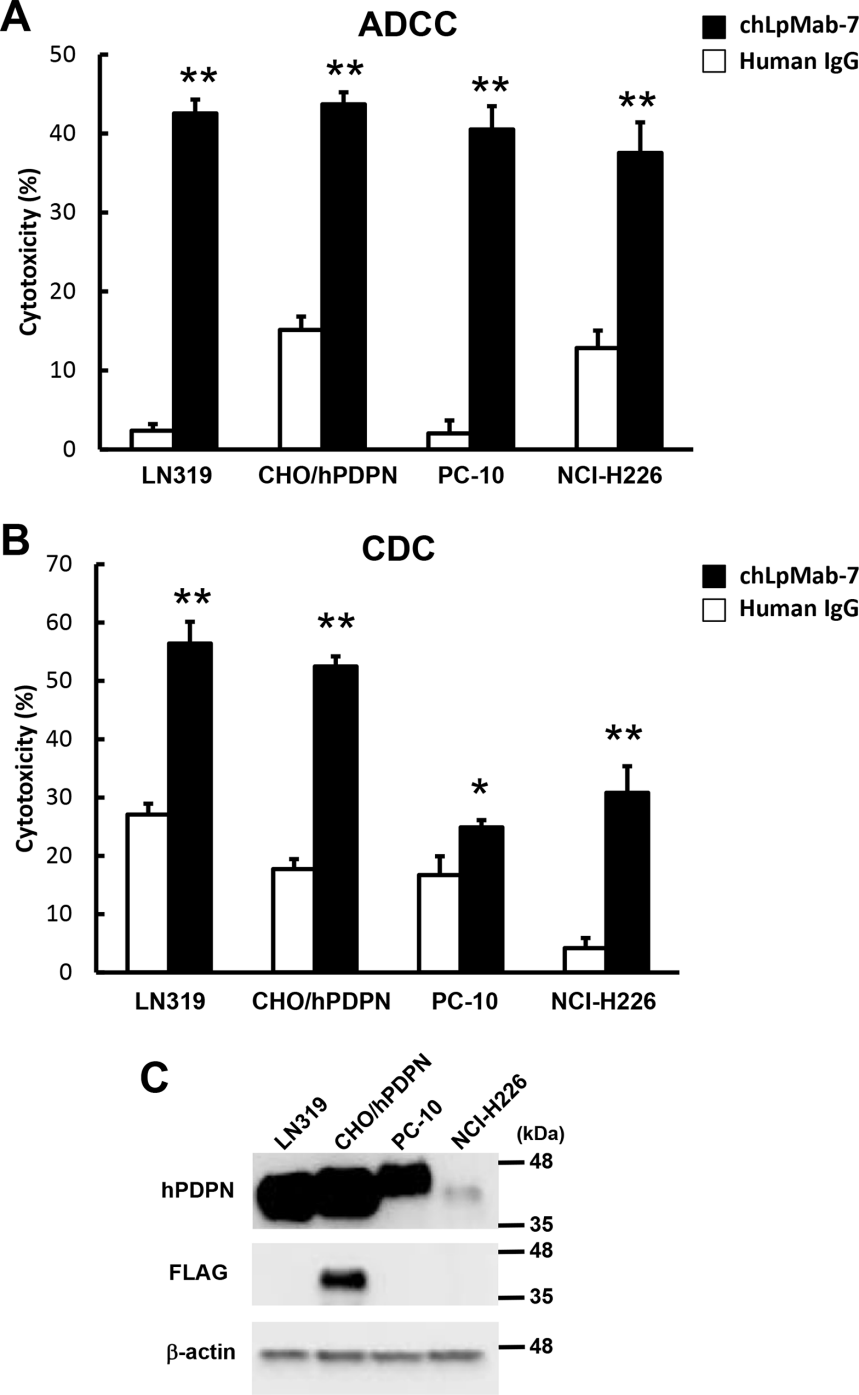
ADCC and CDC activities of chLpMab-7 **A.** ADCC activities induced by human MNC against hPDPN-expressing cell lines were determined using a 6 h ^51^Cr release assay at the E/T ratio of 100 in the presence of 1 μg/ml chLpMab-7 and human IgG. **B.** CDC activities against hPDPN-expressing cell lines were demonstrated by ^51^Cr release assay. *P < 0.05, **P < 0.01 versus control (values are means ± SEM). **C.** Western-blot analysis. Total cell lysates were electrophoresed on 5–20% polyacrylamide gels and transferred onto a PVDF membrane. After blocking, the membrane was incubated with 1 μg/ml mAbs (NZ-1 against hPDPN, 1E5 against FLAG tag, AC-15 against β-actin) and then with peroxidase-conjugated secondary antibodies; the membrane was analyzed using a Sayaca-Imager.

To develop targeted therapy against hPDPN, we assessed whether chLpMab-7 can induce ADCC against PDPN-expressing cell lines mediated by human mononuclear cells (MNC), including NK cells and macrophages, as effector cells. As shown in Figure 5A, ADCC by chLpMab-7 was observed against LN319, PC-10, NCI-H226, and CHO/hPDPN cells. ChLpMab-7 showed high ADCC activity against LN319 glioblastoma cells (42.6%), CHO/hPDPN cells (43.7%), PC-10 lung cancer cells (40.5%), and NCI-H226 malignant mesothelioma cells (37.5%). We next investigated whether chLpMab-7 can induce CDC against hPDPN-expressing cell lines (Figure 5B). ChLpMab-7 showed high CDC activity against LN319 glioblastoma cells (56.4%) and CHO/hPDPN cells (52.5%) but only moderate CDC activity against PC-10 lung cancer cells (24.9%) and NCI-H226 malignant mesothelioma cells (30.8%). Western blot analysis showed that LN319 and CHO/hPDPN cells expressed higher levels of hPDPN compared with PC-10 and NCI-H226 cells (Figure 5C).

### Anti-tumor effect of chLpMab-7 on primary tumor development and spontaneous lung metastasis in nude mice inoculated with hPDPN-expressing cells

**Figure 6:**
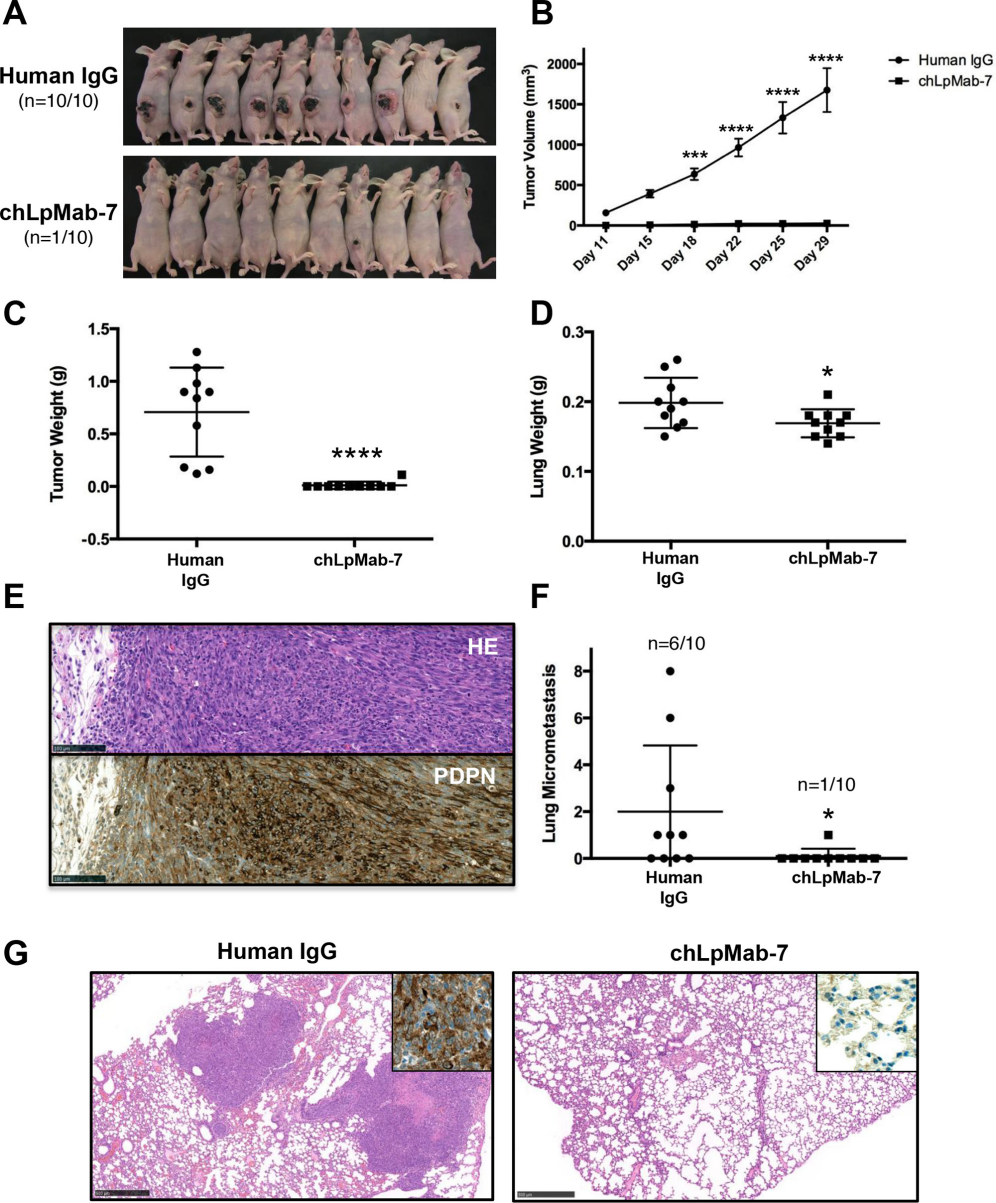
Anti-tumor effect of chLpMab-7 on primary tumor development and spontaneous lung metastasis in nude mice inoculated with hPDPN-expressing cells CHO/hPDPN cells (3 × 10^6^ cells/100 μl) were inoculated subcutaneously into BALB/c nude mice. After one day, 100 μg of chLpMab-7 or human IgG antibodies was injected into the peritoneal cavity of mice. The antibodies were injected once per week for three weeks (human IgG group: *n* = 10; chLpMab-7 group: *n* = 10). **A.** Comparison of the tumor size and tumor incidence in nude mice (day 30). **B.** Primary tumor growth in human IgG- or chLpMab-7-treated mice. ***p < 0.001; ****p < 0.0001 by two-way ANOVA. **C.** Primary tumor weight of human IgG or chLpMab-7 treated mice. ****p < 0.0001. **D.** Lung weight of human IgG- or chLpMab-7 treated mice. *p < 0.05. **E.** HE staining and immunostaining with NZ-1 against the primary tumor formed by injecting human IgG after inoculating CHO/hPDPN cells subcutaneously. Bars, 100 μm. **F.** Spontaneous metastasis of CHO/hPDPN cells was inhibited by chLpMab-7 treatment. Incidence and number of metastases per lung following s.c. inoculation. *p < 0.05. **G.** HE staining of the lung from human IgG or chLpMab-7-treated mice. The inset shows the higher magnification image of immunostaining of the lungs for hPDPN. Bars, 500 μm.

To investigate the anti-tumor activity of chLpMab-7 on primary tumor growth *in vivo*, CHO/hPDPN cells were implanted subcutaneously into the flanks of nude mice. ChLpMab-7 or control human IgG antibodies were injected into the peritoneal cavity of mice once a week for three weeks (*n* = 10 each). Tumor formation was observed in all human IgG group mice (tumor incidence: 100%: 10/10; Figure 6A). In contrast, chLpMab-7 dramatically reduced the tumor development (tumor incidence: 10%: 1/10). Tumor volume and tumor weight were significantly reduced by chLpMab-7 treatment (Figure 6B and 6C). Furthermore, lung weight was reduced by chLpMab-7 treatment (Figure 6D). The primary tumor formation was confirmed by HE staining and immunostaining (Figure 6E). Immunohistochemistry with an anti-hPDPN antibody (NZ-1) showed membranous staining in tumor cells. Furthermore, HE staining revealed the spontaneous formation of lung micrometastases, which was observed in 6 of 10 control mice (60%) and was remarkably reduced to 10% (1/10) by chLpMab-7 treatment (Figure 6F and 6G). Lung micrometastasis foci were confirmed by immunostaining against hPDPN. The expression of hPDPN was maintained in the lung metastasis (Figure 6G, inset). These results indicate that the administration of chLpMab-7 inhibited the primary tumor growth of CHO/hPDPN cells and reduced spontaneous lung metastasis.

### Anti-tumor effect of chLpMab-7 against endogenous hPDPN-expressing cells

**Figure 7:**
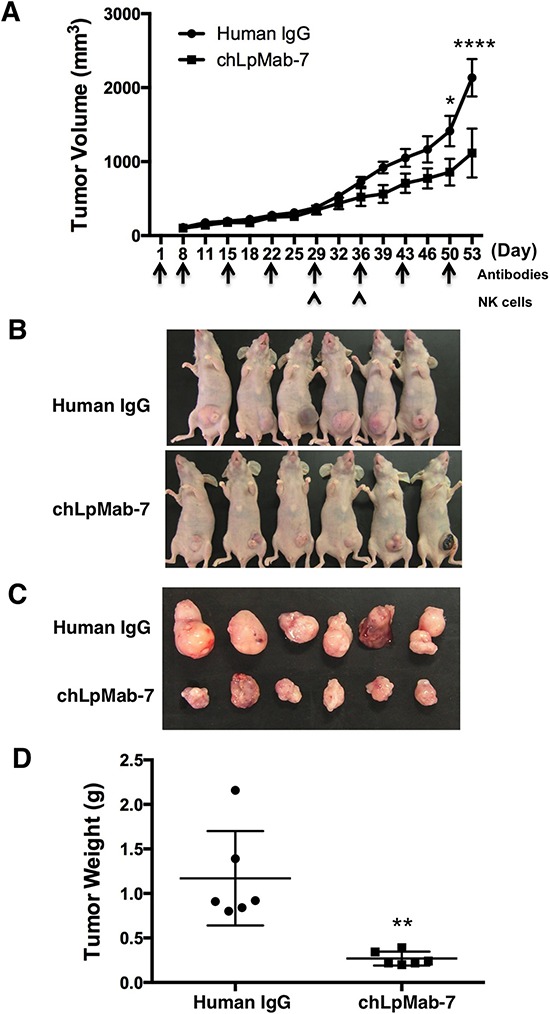
Anti-tumor effect of chLpMab-7 against PC-10 xenografts PC-10 cells (5 × 10^6^ cells/100 μl) were inoculated subcutaneously into BALB/c nude mice. After one day, 100 μg of chLpMab-7 or human IgG antibodies was injected into the peritoneal cavity of mice. The antibodies were injected once per week (human IgG group: *n* = 6; chLpMab-7 group: *n* = 6). Human NK cells were injected around the tumors at Day 29 and Day 36. **A.** Primary tumor growth in human IgG- or chLpMab-7-treated mice. *p < 0.05; ****p < 0.0001 by two-way ANOVA. **B.** Comparison of the tumor size and tumor incidence in nude mice (Day 53). **C.** Gross morphology of the xenograft tumor. **D.** Primary tumor weight of human IgG- or chLpMab-7 treated mice. ** *p* < 0.01.

To further investigate the anti-tumor effect of chLpMab-7 on endogenous hPDPN-expressing cells *in vivo*, PC-10 cells were implanted subcutaneously into the flanks of nude mice. ChLpMab-7 or control human IgG antibodies were injected into the peritoneal cavity of mice once a week (*n* = 6 each). Tumor formation was evenly observed in both groups of mice for 29 days, although chLpMab-7 was injected four times, indicating that the CDC activity of chLpMab-7 was not sufficient to inhibit tumor growth in the PC-10 xenograft model (Figure 7A). ChLpMab-7 does not exhibit ADCC activity in the mouse model; therefore, human NK cells were injected around the tumors at Day 29 and Day 36 to induce ADCC activity together with i.p. injection of chLpMab-7. Tumor volume was significantly reduced by chLpMab-7 treatment after two injections of NK cells, indicating that the ADCC activity of chLpMab-7 was induced effectively (Figure 7A, 7B, and 7C). Tumor weight was significantly reduced by chLpMab-7 treatment (Figure 7D). These results indicate that the administration of chLpMab-7 inhibited the primary tumor growth of PC-10 cells.

### Suppression of experimental lung metastasis by chLpMab-7

**Figure 8:**
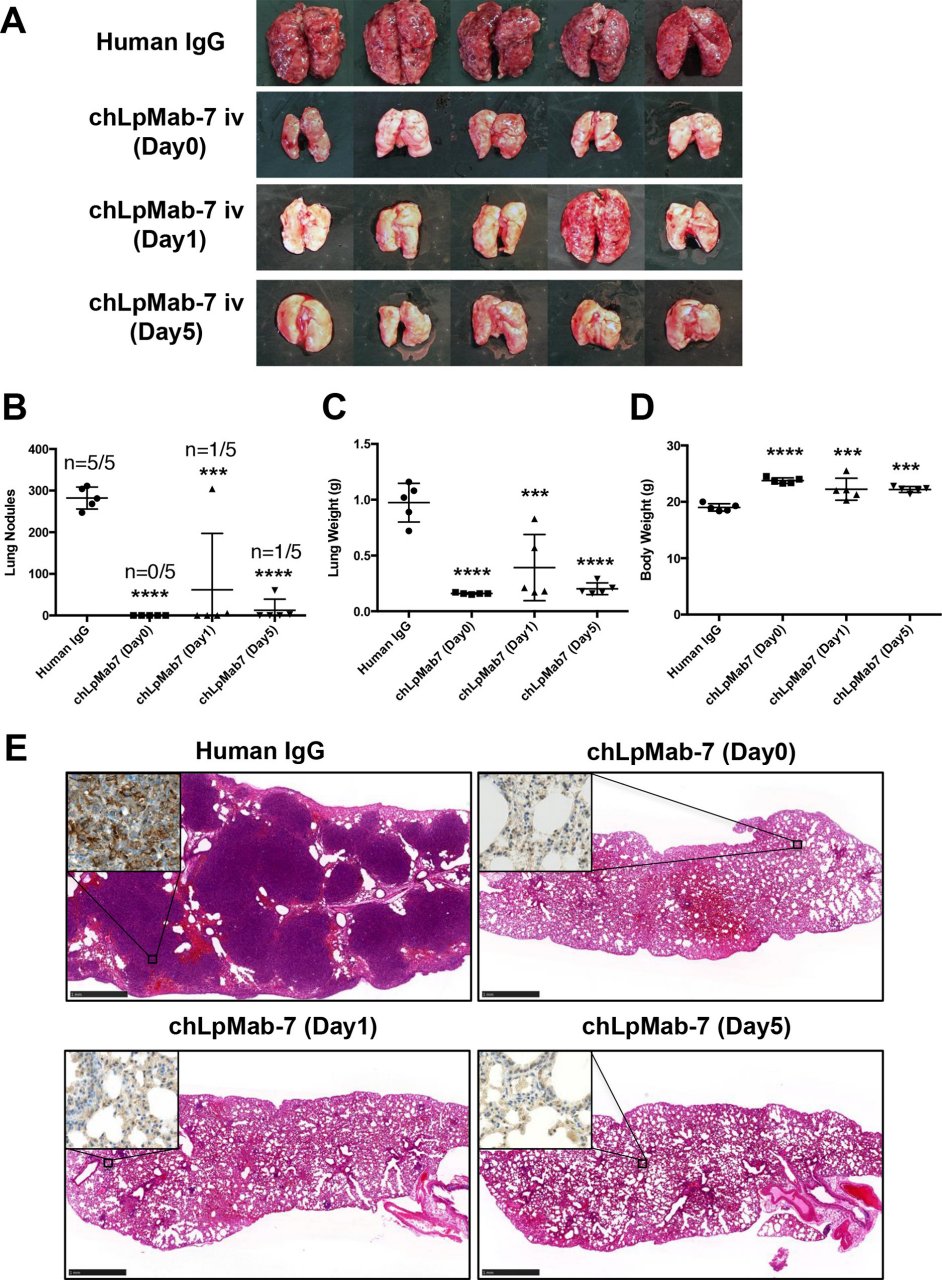
Suppression of experimental lung metastasis by chLpMab-7 CHO/hPDPN cells (5 × 10^5^ cells/100 μl) were injected intravenously into nude mice. Animals were assigned to 4 groups, which received chLpMab-7 or human IgG concomitantly with the cell injection (Day 0), chLpMab-7 treatment 1 day after tumor cell injection (Day 1), or chLpMab-7 treatment 5 days after cell injection (Day 5). **A.** Visible lung metastasis. **B.** Incidence and number of visible metastases per lung. **C.** Lung weight. **D.** Body weight. ***p < 0.001; ****p < 0.0001. **E.** HE staining of the lung from each mouse. The inset shows the higher magnification image of immunostaining of the lungs for hPDPN. Bars, 1 mm.

We next investigated whether chLpMab-7 antibodies could suppress hPDPN-induced pulmonary metastasis in an experimental metastasis model. The injection of CHO/hPDPN cells led to the development of multiple lung metastatic foci (5/5: 100%, Figure 8A). ChLpMab-7 antibodies were administered concomitantly with the cell injection (Day 0) or one day (Day 1) or 5 days (Day 5) after cell injection. As a result, visible lung metastases were dramatically reduced by the chLpMab-7 treatment (Figure 8A). Lung metastasis was completely blocked by the chLpMab-7 treatment concomitantly with the cell injection (Day 0, Figure 8B). Treatment with chLpMab-7 after the cell injection (Day 1 or Day 5) also blocked metastasis. Lung weight was significantly reduced by chLpMab-7 treatment (Figure 8C). In addition, body weight was significantly improved in all chLpMab-7-treated groups (Figure 8D). HE staining revealed that many metastatic foci were present in the lungs of the human IgG group, whereas much fewer metastatic foci were observed in the chLpMab-7 groups (Figure 8E). Immunostaining for hPDPN confirmed the presence of lung metastasis (Figure 8E, inset).

## DISCUSSION

Although anti-hPDPN mAbs with high sensitivity and specificity are required to investigate the physiological function of hPDPN in cancers and normal tissues, almost all anti-hPDPN mAbs have been developed against the PLAG domains of hPDPN because the neutralizing activities of anti-hPDPN mAbs have been reported to be important for targeting therapy against hPDPN [9, 35, 37]. Using anti-hPDPN mAbs recognizing the PLAG domain, the same glycan profile, such as the sialyl-core1 structure, was observed by antibody-overlay lectin microarray using anti-PLAG mAbs [9, 38]. In contrast, LpMab-7, an anti-non-PLAG hPDPN mAb, can be used for detecting different glycan profiles of hPDPN, including the poly LacNAc structure (Figure 1C), indicating that LpMab-7 may be advantageous for analyzing the novel pathophysiological function of hPDPN in cancers and normal tissues.

We recently established a platform to produce cancer-specific mAbs (CasMabs) [31]. Briefly, the membranous proteins were transfected into cancer cell lines, which express cancer-specific glycans. For hPDPN expression, we used LN229 glioma cells, which exhibit cancer-type glycan patterns, including aberrant sialylation and highly sulfated polylactosamine. We immunized mice with LN229/hPDPN cells and developed LpMab-2, a CasMab. At the same time, we could produce many other anti-hPDPN mAbs. Some of these mAbs are glycopeptide-recognizing mAbs (GpMabs), such as LpMab-3 and LpMab-9, and others are highly sensitive mAbs, such as LpMab-7. We named this advantageous mAb-producing method the “CasMab method.” LpMab-7 was shown to be more sensitive than NZ-1 and D2–40 by immunohistochemistry [39].

We previously showed that the epitope of LpMab-7 is Arg79-Leu83 of hPDPN and that Ile80, Glu81, and Leu83 are the most important amino acids [32]. In the present study, we also demonstrated that the epitope of LpMab-7 is entirely different from that of NZ-1, a neutralizing mAb against the PLAG domain, using an inhibition assay. Unfortunately, the subclass of LpMab-7 is mouse IgG_1_, which does not induce ADCC and CDC; therefore, LpMab-7 was converted to the human IgG_1_ subclass.

The binding affinity of chLpMab-7 was better than that of the original mouse LpMab-7, although those variable regions are the same. Our previous study demonstrated that rat-human chimeric NZ-1, which was designated as NZ-8, showed a lower affinity compared with NZ-1: the *K_D_* of NZ-1 was determined to be 7.2 × 10^−9^ M by flow cytometry or 4.1 × 10^−10^ M by ELISA, whereas the *K_D_* of NZ-8 was determined to be 7.6 × 10^−8^ M by flow cytometry or 1.8 × 10^−9^ M by ELISA [40], although the same constant regions of heavy chain (human IgG_1_) and light chain (kappa) were used for both chLpMab-7 and NZ-8. We have characterized many human-mouse chimeric mAbs and compared their affinities with those of the original mouse mAbs; however, almost the same binding affinities have been obtained (data not shown). Therefore, we have thus far been unable to determine the reason why the binding affinity of chLpMab-7 improved after the conversion of the constant region from mouse IgG_1_ to human IgG_1_.

ChLpMab-7 not only possesses high binding affinity but also showed high ADCC and CDC activities against hPDPN-expressing cancer cells, suggesting that chLpMab-7 offers more advantages for antibody-based molecular targeting therapy. LpMab-7 showed high ADCC activities against all cancer cells; in contrast, it showed higher CDC activities against the LN319 glioblastoma cell line than against PC-10 lung cancer cells and NCI-H226 malignant mesothelioma cells, most likely because i) LpMab-7 was produced by immunizing mice with LN229/hPDPN glioblastoma cells; ii) the post-translational modification, including the glycosylation of hPDPN, is different among cancer cells [31]; or iii) the expression level of hPDPN in LN319 is much higher than that of the other cell lines. Indeed, Western blot analysis showed that LN319 and CHO/hPDPN cells express higher levels of hPDPN compared with PC-10 and NCI-H226 cells (Figure 5C). Although PC-10 cells express a much higher level of hPDPN than NCI-H226 cells (Figure 5C), lower CDC activity was observed in PC-10 cells compared with NCI-H226 cells, indicating that ADCC/CDC activities may depend on several interrelated factors. We investigated whether chLpMab-7 exerts effects on cancer cell growth, sphere forming property, adhesion, and migration *in vitro*. However, chLpMab-7 produced no effect on cancer cell growth (Supplementary Figure S1A), sphere forming property (Supplementary Figure S1B), adhesion (Supplementary Figure S2), or migration *in vitro* (Supplementary Figure S3).

It has been demonstrated that all anti-hPDPN mAbs suppress hPDPN-induced pulmonary metastasis by inhibiting platelet aggregation [13, 33, 41, 42]. Previous studies showed that not only whole IgGs but also F(ab’)_2_ fragments almost completely suppressed the formation of pulmonary metastasis by hPDPN-expressing cells. In contrast, whole IgGs, such as NZ-1 and NZ-8, clearly demonstrated ADCC and CDC activities and inhibited the growth of hPDPN-expressing cells *in vivo*, indicating that the suppression of pulmonary metastasis might be dependent on both neutralizing activity and ADCC/CDC activities. However, it has not been determined that ADCC or CDC activities are sufficient for suppressing hPDPN-induced pulmonary metastasis because almost all anti-hPDPN mAbs have been produced against the anti-PLAG domain (amino acids 29–54) of hPDPN. In this study, we investigated whether an anti-non-PLAG hPDPN domain mAb can inhibit the pulmonary metastasis of hPDPN-expressing cells in a neutralization-independent manner. The injection of chLpMab-7, which recognizes Arg79-Leu83 of hPDPN, significantly blocked the formation of pulmonary metastasis even 5 days after the injection of cancer cells, suggesting that the pulmonary metastasis of hPDPN-expressing cells can be inhibited in a neutralization-independent manner. In mouse models, chLpMab-7 can exhibit only CDC activity because mouse NK cells or macrophages do not bind to human IgG_1_. Although chLpMab-7 demonstrated a high anti-tumor activity in both xenograft and experimental metastasis models of CHO/hPDPN cells, only chLpMab-7 did not show an anti-tumor effect in a PC-10 xenograft. After human NK cell injection around a PC-10 xenograft together with i.p. injection of chLpMab-7, the tumor volume was significantly reduced, indicating that both ADCC and CDC activities were much more toxic against hPDPN-expressing tumors.

In this study, we did not seek to minimize the importance of the PDPN-CLEC-2 interaction for the tumor development and cancer metastasis. PDPN can bind to CLEC-2, the only endogenous receptor of PDPN, and induce platelet aggregation. Several growth factors, such as TGF-β and PDGF from activated platelets, may contribute to the tumor development in the microenvironment. Therefore, neutralizing mAbs might also be effective by targeting PDPN-expressing tumors.

## MATERIALS AND METHODS

### Cell lines and stable transfectants

Chinese hamster ovary (CHO)-K1, LN229, NCI-H226, HEK-293T, and Met-5A cells were obtained from the American Type Culture Collection (ATCC, Manassas, VA). Human lymphatic endothelial cells (LECs) were obtained from Cambrex (Walkersville, MD). PC-10 cells were purchased from Immuno-Biological Laboratories Co., Ltd. (Gunma, Japan). The human glioblastoma cell line LN319 was donated by Dr. Webster K. Cavenee (Ludwig Institute for Cancer Research, San Diego, CA). CHO/hPDPN and LN229/hPDPN were established as described previously [2, 31]. CHO/hPDPN, PC-10, and NCI-H226 cells were cultured in RPMI 1640 medium (Nacalai Tesque, Inc., Kyoto, Japan) supplemented with 10% heat-inactivated fetal bovine serum (FBS; Thermo Fisher Scientific Inc., Waltham, MA), 2 mM _L_-glutamine (Thermo Fisher Scientific Inc.), 100 units/ml penicillin, 100 μg/ml streptomycin, and 25 μg/ml amphotericin B (Nacalai Tesque, Inc.) at 37°C in a humidified atmosphere of 5% CO_2_ and 95% air. Geneticin (0.5 mg/ml; Wako Pure Chemical Industries, Ltd., Osaka, Japan) was added for CHO/hPDPN cells. HEK-293T, LN229, LN229/hPDPN, LN319, and Met-5A cells were cultured in Dulbecco's Modified Eagle's Medium (DMEM) (Nacalai Tesque, Inc.) supplemented with 10% heat-inactivated FBS, 2 mM _L_-glutamine, 100 units/ml penicillin, 100 μg/ml streptomycin, and 25 μg/ml amphotericin B at 37°C in a humidified atmosphere of 5% CO_2_ and 95% air. MITO + serum extender (Thermo Fisher Scientific Inc.) was added for Met-5A cells. Geneticin (0.5 mg/ml) was added for LN229/hPDPN cells. LEC cells were cultured in endothelial cell medium EGM-2MV supplemented with 5% FBS (Cambrex).

### Animals

Female BALB/c nude (nu/nu) mice (5–7-week old) were purchased from Charles River Japan, Inc. (Kanagawa, Japan). Animals were housed under pathogen-free conditions. The Animal Care and Use Committees of the University of Tokyo approved the animal experiments described herein.

### Antibodies

LpMab-7, a mouse anti-hPDPN mAb (IgG_1,_ kappa), was developed as described previously [31]. Human IgGs were purchased from Beckman Coulter, Inc. (Fullerton, CA). For the generation of mouse-human chimeric anti-hPDPN (chLpMab-7), the appropriate V_H_ and V_L_ cDNAs of a mouse LpMab-7 antibody and C_H_ and C_L_ of human IgG_1_ were subcloned into the pCAG-Ble or pCAG-Neo vectors (Wako Pure Chemical Industries, Ltd.), respectively. Antibody expression vectors were transfected into CHO-K1 cells using Lipofectamine LTX reagent (Thermo Fisher Scientific Inc.). Stable transfectants of CHO-K1/chLpMab-7 were selected by culturing the transfectants in medium containing 0.5 mg/ml geneticin and 0.5 mg/ml zeocin (InvivoGen, San Diego, CA). NZ-1, a rat anti-hPDPN mAb (IgG_2a_, lambda) was produced as described previously [9]. 1E5, a mouse anti-FLAG mAb, and AC-15, a mouse anti-β-actin mAb, were purchased from Wako Pure Chemical Industries, Ltd., and Sigma-Aldrich Corp. (St. Louis, MO), respectively.

### Neutralization assays of PDPN and CLEC-2

Inhibition assays were performed using enzyme-linked immunosorbent assay (ELISA). Purified hPDPN-Fc was immobilized on Nunc Maxisorp 96-well immunoplates (Thermo Fisher Scientific Inc.) at 1 μg/ml for 30 min. After blocking with SuperBlock T20 (PBS) Blocking Buffer, LpMab-7 or NZ-1 was added at 1 μg/ml for 30 min. The plates were incubated with biotinylated CLEC-2-Fc (1 μg/ml) followed by 1:1000-diluted peroxidase-conjugated streptavidin (GE Healthcare, Piscataway, NJ). The enzymatic reaction was conducted with a 1-Step Ultra TMB-ELISA (Thermo Fisher Scientific Inc.). The optical density was measured at 655 nm using an iMark microplate reader (Bio-Rad Laboratories Inc., Philadelphia, PA).

### Lectin microarray

CHO/hPDPN cells were solubilized using 1% Triton-X100 in PBS (PBST), and hPDPN was purified using a FLAG-tag system (Sigma-Aldrich Corp.). Then, 100 μl of purified hPDPN (31.25–2000 ng/ml) was applied to a lectin array (LecChip ver1.0; GlycoTechnica, Hokkaido, Japan), including triplicate spots of 45 lectins in each of seven divided incubation baths on the glass slide. After incubation at 20°C for 17 h, 4 μl of human IgG (5 mg/ml; Sigma-Aldrich Corp.) was applied to each well. After incubation at 20°C for 30 min, the glass slide was washed three times with PBST; 60 μl of biotinylated LpMab-7 and NZ-1 (1 μg/ml) in PBS were applied to the array and incubated at 20°C for 3 h. After washing three times with PBST, Cy3-labeled streptavidin (Thermo Fisher Scientific Inc.) was added to the array and incubated at 20°C for 30 min. The glass slide was scanned using a GlycoStation Reader 1200 (GlycoTechnica). Abbreviation of lectins: GNA, *Galanthus nivalis* agglutinin; HHL, *Hippeastrum hybrid* lectin; ACG, *Agrocybe cylindracea* galectin; TxLCI, *Tulipa gesneriana* lectin; BPL, *Bauhinia purpurea alba* lectin; TJA-II, *Trichosanthes japonica* agglutinin; EEL, *Euonymus europaeus* lectin; ABA, *Agaricus bisporus* agglutinin; LEL, *Lycopersicon esculentum* lectin; STL, *Solanum tuberosum* lectin; UDA, *Urtica dioica* agglutinin; PWM, *Pokeweed* mitogen; PNA, peanut agglutinin; WFA, *Wisteria floribunda* agglutinin; ACA, *Amaranthus caudatus* agglutinin; MPA, *Maclura pomifera* agglutinin; HPA, *Helix pomatia* agglutinin; VVA, *Vicia villosa* agglutinin; DBA, *Dolichos biflorus* agglutinin; SBA, Soybean agglutinin; PTL I, *Psophocarpus tetragonolobus* lectin I; MAH, *Maackia amurensis* hemagglutinin; WGA, wheat germ agglutinin; GSL-I, *Griffonia simplicifolia* lectin I.

### Flow cytometry

Cells were harvested by brief exposure to 0.25% Trypsin/1 mM EDTA (Nacalai Tesque, Inc.). After washing with PBS, the cells were treated with LpMab-7 and chLpMab-7 (1 μg/ml) for 30 min at 4°C followed by incubation with Oregon green-conjugated anti-mouse IgG or FITC-conjugated anti-human IgG (Thermo Fisher Scientific Inc.). Fluorescence data were collected using a Cell Analyzer EC800 (Sony Corp., Tokyo, Japan).

### Determination of binding affinity by flow cytometry

Binding affinity was determined as described previously [43]. Briefly, cells (2 × 10^5^) were resuspended in 100 μl of serially diluted antibodies (0.02–100 μg/ml), followed by secondary antibodies (Thermo Fisher Scientific Inc.). Fluorescence data were collected using a Cell Analyzer EC800. The apparent dissociation constants (*K_D_*) were obtained by fitting the binding isotherms using built-in one-site binding models in Prism software (GraphPad Software, San Diego, CA).

### Preparation of effector cells

Human peripheral blood mononuclear cells (MNCs) were obtained from the peripheral blood of healthy donors as described previously [44]. The human study was approved by the ethics committee of the University of Tokushima, and written informed consent was obtained from all study participants. Human natural killer cells were purchased from Takara Bio Inc. (Shiga, Japan).

### Antibody-dependent cellular cytotoxicity (ADCC) assay

ADCC was evaluated using a ^51^Cr release assay as described previously [45]. Target cells were labeled with 0.1 μCi of ^51^Cr-sodium chromate at 37°C for 1 h. ^51^Cr-labeled target cells were placed in 96-well plates in triplicate, to which effector cells and antibodies (chLpMab-7 and human IgG) were added. After a 6 h incubation, the radioactivity present in 100 μl of supernatant was measured in a gamma counter (PerkinElmer, Waltham, MA). The percentage of cytotoxicity was calculated using the following formula: % Specific lysis = (E – S)/(M – S) × 100, where E is the release in the test sample, S is the spontaneous release, and M is the maximum release.

### Complement-dependent cytotoxicity (CDC) assay

CDC was determined by ^51^Cr release assay [45]. Target cells were labeled with 0.1 μCi of ^51^Cr-sodium chromate at 37°C for 1 h. After incubation, the cells were washed with medium three times. ^51^Cr-labeled cells were added to 96-well plates and incubated with baby rabbit complement and chLpMab-7 and human IgG for 6 h. ^51^Cr release in the supernatant from each well (100 μl) was measured using a gamma counter. The percentage of cytotoxicity was calculated as above.

### Western blot analyses

Cell lysates (10 μg) were boiled in SDS sample buffer (Nacalai Tesque, Inc.). The proteins were electrophoresed on 5–20% polyacrylamide gels (Wako Pure Chemical Industries Ltd.) and transferred onto a PVDF membrane (EMD Millipore Corp., Billerica, MA). After blocking with 4% skim milk (Nacalai Tesque, Inc.), the membrane was incubated with primary antibodies, then with peroxidase-conjugated secondary antibodies (Dako; 1/1000 diluted), and developed with Pierce Western Blotting Substrate Plus (Thermo Fisher Scientific Inc.) or ImmunoStar LD Chemiluminescence Reagent (Wako Pure Chemical Industries Ltd.) using a Sayaca-Imager (DRC Co. Ltd., Tokyo, Japan).

### Anti-tumor activity of anti-hPDPN antibodies on CHO/hPDPN cells

CHO/hPDPN cells were suspended using trypsin, washed and suspended with PBS, adjusted to a density of 3 × 10^7^ cells/ml, and inoculated subcutaneously into BALB/c nude mice at a dose of 100 μl/animal. After one day, 100 μl of 1 mg/ml chLpMab-7 or human IgG were respectively injected into the peritoneal cavity of mice, and the antibodies were injected once per week for three weeks (control group: *n* = 10; chLpMab-7 group: *n* = 10). The tumor diameter was measured at intervals of three to four days and was calculated using the following formula: tumor volume = W^2^ × *L*/2, where *W* = short diameter and *L* = long diameter. On Day 30 after cell implantation, the mice were euthanized and the lungs and primary tumor tissues harvested for hematoxylin and eosin (HE) staining.

### Immunohistochemical analyses

All tissue samples were fixed in formalin and embedded in paraffin, and sections were cut at 3 μm thickness. Immunostaining was performed according to standard techniques using an autostainer (BenchMark XT; Ventana Medical Systems, Inc., Tucson, AZ, USA). The anti-hPDPN antibody NZ-1 [9] was used at a concentration of 1 μg/ml, and secondary biotin-conjugated anti-rat antibody (Vector Laboratories, Peterborough, UK) was used at 1:200 dilution.

### Anti-tumor activity of anti-hPDPN antibodies on PC-10 cells

PC-10 cells were suspended using trypsin, washed and suspended with PBS, adjusted to a density of 5 × 10^7^ cells/ml, and inoculated subcutaneously into BALB/c nude mice at a dose of 100 μl/animal. After one day, 100 μl of 1 mg/ml chLpMab-7 or human IgG were respectively injected into the peritoneal cavity of mice, and the antibodies were injected once per week for 50 days (control group: *n* = 6; chLpMab-7 group: *n* = 6). Human NK cells (5 × 10^5^) were injected around the tumors at Day 29 and Day 36. The tumor diameter was measured at intervals of three to four days and calculated using the same formula as above. On Day 53 after cell implantation, the mice were euthanized and primary tumor tissues were harvested.

### Experimental lung metastasis

CHO/hPDPN cells were harvested, washed, and resuspended in PBS (5 × 10^6^ cells/ml). The cells were incubated with chLpMab-7 or human IgG and inoculated intravenously (5 × 10^5^ cells/mouse) into the lateral tail vein of female BALB/c-nu/nu mice. In some groups, chLpMab-7 antibodies were inoculated either 1 day or 5 days after cell injection into the tail vein. After 17 days, the mice were euthanized and the surface lung metastatic foci was counted and measured. The lung tissues from CHO/hPDPN-bearing mice were processed for HE staining.

### Statistical analyses

All data are shown as means ± SEM. Student's *t* test, Mann-Whitney *U*-test, one-way ANOVA followed by Tukey-Kramer multiple comparisons, and two-way ANOVA were performed as indicated. *P* values < 0.05 were considered statistically significant. All statistical tests were two-sided.

## SUPPLEMENTARY METHODS AND FIGURES


