# Supplementary material for: The chimeric antibody chLpMab-7 targeting human podoplanin suppresses pulmonary metastasis via ADCC and CDC rather than via its neutralizing activity
Source: Oncotarget. 2015 Sep 25;6(34):36003–18. doi: 10.18632/oncotarget.5339 (PMC4742157; doi:10.18632/oncotarget.5339)
Supplement: Supplementary file 1 [file oncotarget-06-36003-s001.pdf]

# SUPPLEMENTARY METHODS AND FIGURES

## *In vitro* growth assay

Cells were treated with 10 μg/ml human IgG or chLpMab-7, and the effect on cell growth was examined. For the monolayer culture growth, CHO/hPDPN cells ($1 \times 10^4$) were seeded into a 6-well plate, and cell confluence was monitored with JuLI Stage Automated cell imaging system and software (NanoEnTek, Seoul, Korea). To examine the three-dimensional proliferation of cells, $10^3$ cells were seeded into a round-bottom 96-well plate (PrimeSurface 96U, Sumitomo Bakelite, Tokyo, Japan), and the diameters of the spheroids were measured for 3 days.

## *In vitro* adhesion assay

Cell adhesion to the culture plate was assessed by monitoring the cell number with JuLI Stage Automated cell imaging system and software. CHO/hPDPN cells ($1 \times 10^5$) were seeded into a 6-well plate with 10 μg/ml human IgG or chLpMab-7 and were incubated for 1 h at 37°C. After changing the medium, the number of attached cells was calculated using JuLI Stage software.

## Migration assay

Wound confluence was monitored using JuLI Stage Automated cell imaging system and software. Wound closure was monitored with cells plated in a 6-well plate every h for 40 h after treatment with 10 μg/ml human IgG or chLpMab-7.

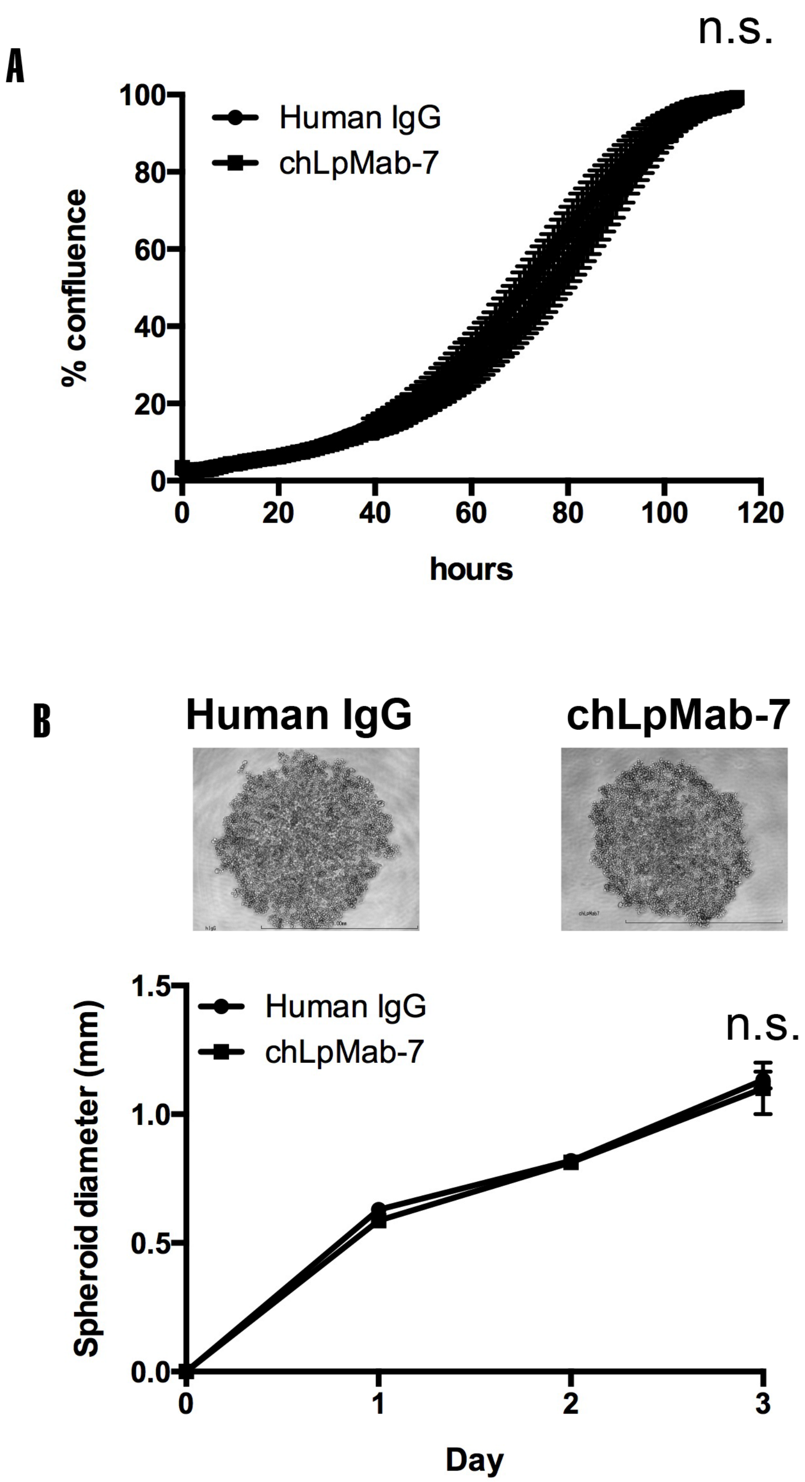


**Supplementary Figure S1: *In vitro* growth assay.** The proliferation of PDPN-expressing cells was not changed by treatment with chLpMab-7. CHO-PDPN cells were treated with 10 µg/ml human IgG or chLpMab-7. **A.** Growth of the cells in monolayer culture. Cells were imaged under culture conditions using JuLI Stage. Data are presented as the mean ± SEM (*n* = 10). **B.** Growth of cells in three-dimensional spheroid culture. Representative images taken at 72 h after cell seeding. Data are presented as the mean ± SEM (*n* = 3). n.s.: not significant.

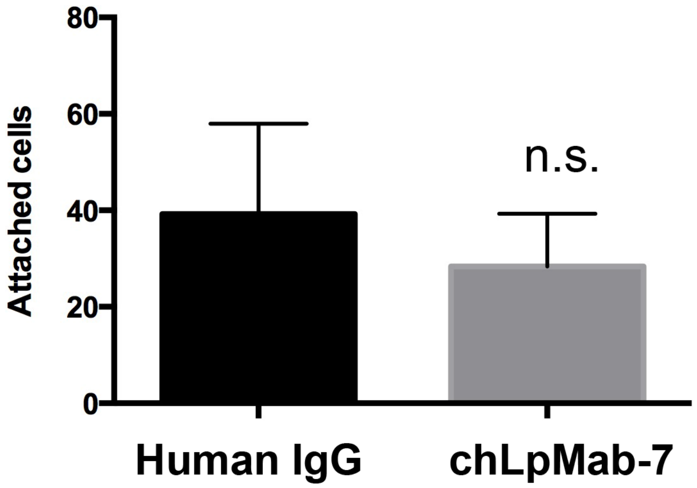


**Supplementary Figure S2: *In vitro* adhesion assay to measure the effect of chLpMab-7 on CHO-PDPN cells.** CHO-PDPN cells were plated in the presence of 10 µg/ml human IgG or chLpMab-7. One hour later, the culture medium was changed and the number of attached cells was measured using JuLI Stage. Data are presented as the mean ± SD ($n$ = 10). n.s.: not significant.

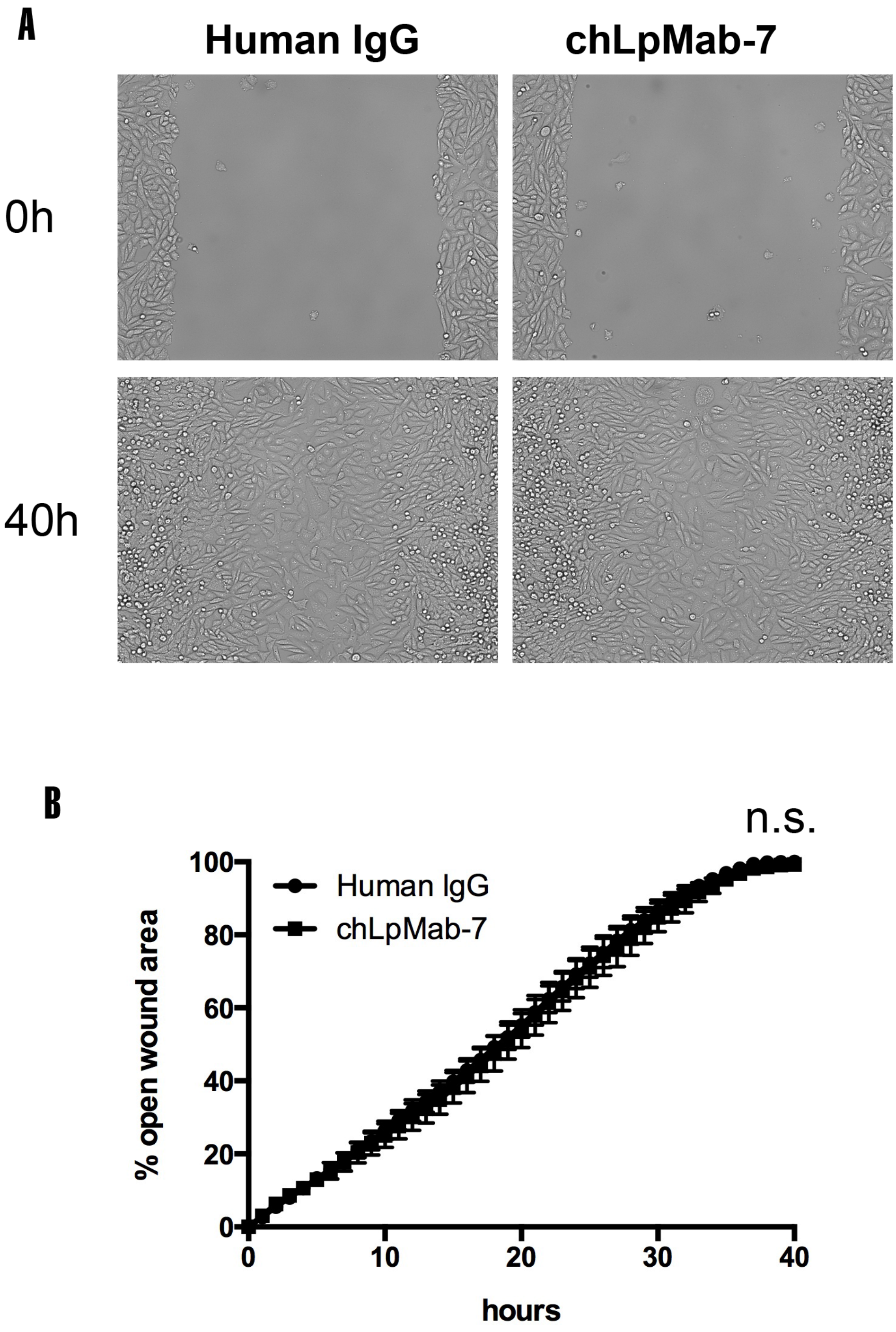


**Supplementary Figure S3: *In vitro* migration assay.** The migration rate of PDPN-expressing cells was not changed by treatment with chLpMab-7. CHO-PDPN cells were grown to confluence and scratched and then treated with 10 µg/ml human IgG or chLpMab-7. The cells were imaged under culture conditions using JuLI Stage. **A.** Representative images taken at 0 h and 40 h. **B.** Wound closure was monitored every hour. Data are presented as the mean ± SEM ($n$ = 6). n.s.: not significant.
